# Association of an impaired GH-IGF-I axis with cardiac wasting in patients with advanced cancer

**DOI:** 10.1007/s00392-024-02400-x

**Published:** 2024-04-08

**Authors:** Ann-Kathrin Fröhlich, Jan Porthun, Khawaja M. Talha, Alessia Lena, Sara Hadzibegovic, Ursula Wilkenshoff, Frederike Sonntag, Anja Nikolski, Luisa Valentina Ramer, Tanja Zeller, Ulrich Keller, Lars Bullinger, Stefan D. Anker, Wilhelm Haverkamp, Stephan von Haehling, Wolfram Doehner, Ursula Rauch, Carsten Skurk, John G. F. Cleland, Javed Butler, Andrew J. S. Coats, Ulf Landmesser, Mahir Karakas, Markus S. Anker

**Affiliations:** 1 Charité – University Medicine Berlin corporate member of Free University Berlin and Humboldt-University Berlin, Berlin, Germany; 2 German Centre for Cardiovascular Research (DZHK), Partner Site Berlin, Berlin, Germany; 3 Berlin Institute of Health Center for Regenerative Therapies (BCRT), Berlin, Germany; 4 Department of Cardiology, Angiology and Intensive Care Medicine Campus Virchow Clinic, German Heart Center Charité, Berlin, Germany; 5 Norwegian University of Science and Technology, Campus Gjøvik, Gjøvik, Norway; 6 Department of Medicine, University of Mississippi Medical Center, Jackson, MS USA; 7 Department of Cardiology, Angiology and Intensive Care Medicine Campus Benjamin Franklin, German Heart Center Charité, Hindenburgdamm 30, 12200 Berlin, Germany; 8 Berlin Institute of Health, Charité – University Medicine Berlin, Berlin, Germany; 9 University Center of Cardiovascular Science, University Medical Centre Hamburg-Eppendorf, Hamburg, Germany; 10 Clinic for Cardiology, University Heart and Vascular Centre Hamburg, University Medical Centre Hamburg-Eppendorf, Hamburg, Germany; 11 German Centre for Cardiovascular Research, Partner Site HH/Kiel/HL, Hamburg, Germany; 12 Department of Hematology, Oncology and Cancer Immunology, Charité – University Medicine Berlin, Campus Benjamin Franklin, Berlin, Germany; 13 German Cancer Consortium (DKTK), Partner Site Berlin, a partnership between DKFZ and Charité-Universitätsmedizin Berlin, Berlin, Germany; 14 Max Delbrück Center, Berlin, Germany; 15 Department of Hematology, Oncology, and Tumor Immunology, Charité – University Medicine Berlin corporate member of Free University Berlin and Humboldt University Berlin, Berlin, Germany; 16 Department of Cardiology Campus, Virchow Clinic of German Heart Center Charité, Charité – University Medicine Berlin, Berlin, Germany; 17 Department of Cardiology and Pneumology, University of Göttingen Medical Center, Göttingen, Germany; 18 German Centre for Cardiovascular Research (DZHK), Partner Site Göttingen, Göttingen, Germany; 19 Centre for Stroke Research, Berlin, Charité-Universitätsmedizin, Berlin, Germany; 20 School of Cardiovascular and Metabolic Health, University of Glasgow, Glasgow, UK; 21 Baylor Scott and White Research Institute, Dallas, TX USA; 22 Heart Research Institute, Sydney, Australia; 23 Department of Intensive Care Medicine, University Medical Center Hamburg-Eppendorf, Hamburg, Germany

**Keywords:** Left ventricular mass, Growth hormone, Insulin-like growth factor-I, Cardiology, Cancer, Echocardiography, Cachexia

## Abstract

**Background:**

Growth hormone (GH) resistance is characterized by high GH levels but low levels of insulin-like growth factor-I (IGF-I) and growth hormone binding protein (GHBP) and, for patients with chronic disease, is associated with the development of cachexia.

**Objectives:**

We investigated whether GH resistance is associated with changes in left ventricular (LV) mass (cardiac wasting) in patients with cancer.

**Methods:**

We measured plasma IGF-I, GH, and GHBP in 159 women and 148 men with cancer (83% stage III/IV). Patients were grouped by tertile of echocardiographic LVmass/height^2^ (women, < 50, 50–61, > 61 g/m^2^; men, < 60, 60–74, > 74 g/m^2^) and by presence of wasting syndrome with unintentional weight loss (BMI < 24 kg/m^2^ and weight loss ≥ 5% in the prior 12 months). Repeat echocardiograms were obtained usually within 3–6 months for 85 patients.

**Results:**

Patients in the lowest LVmass/height^2^ tertile had higher plasma GH (median (IQR) for 1^st^, 2^nd^, and 3^rd^ tertile women, 1.8 (0.9–4.2), 0.8 (0.2–2.2), 0.5 (0.3–1.6) ng/mL, *p* = 0.029; men, 2.1 (0.8–3.2), 0.6 (0.1–1.7), 0.7 (0.2–1.9) ng/mL, *p* = 0.003). Among women, lower LVmass was associated with higher plasma IGF-I (68 (48–116), 72 (48–95), 49 (35–76) ng/mL, *p* = 0.007), whereas such association did not exist for men. Patients with lower LVmass had lower log IGF-I/GH ratio (women, 1.60 ± 0.09, 2.02 ± 0.09, 1.88 ± 0.09, *p* = 0.004; men, 1.64 ± 0.09, 2.14 ± 0.11, 2.04 ± 0.11, *p* = 0.002). GHBP was not associated with LVmass. Patients with wasting syndrome with unintentional weight loss had higher plasma GH and GHBP, lower log IGF-I/GH ratio, and similar IGF-I. Overall, GHBP correlated inversely with log IGF-I/GH ratio (women, *r* =  − 0.591, *p* < 0.001; men, *r* =  − 0.575, *p* < 0.001). Additionally, higher baseline IGF-I was associated with a decline in LVmass during follow-up (*r* =  − 0.318, *p* = 0.003).

**Conclusion:**

In advanced cancer, reduced LVmass is associated with increased plasma GH and reduced IGF-I/GH ratio, suggesting increasing GH resistance, especially for patients with wasting syndrome with unintentional weight loss. Higher baseline IGF-I was associated with a decrease in relative LVmass during follow-up.

**Graphical abstract:**

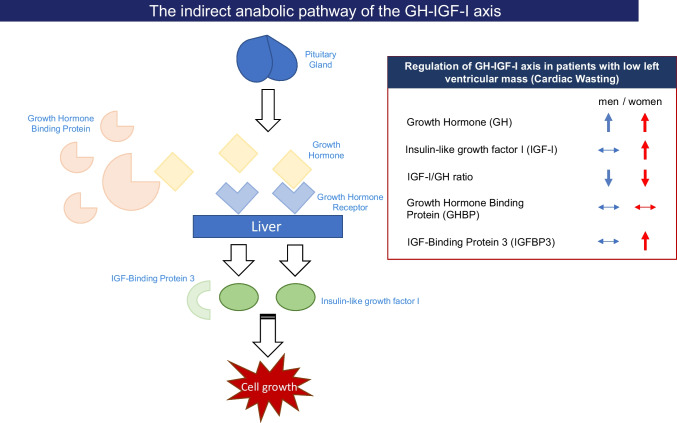

**Supplementary Information:**

The online version contains supplementary material available at 10.1007/s00392-024-02400-x.

## Introduction

**Fig. 1:**
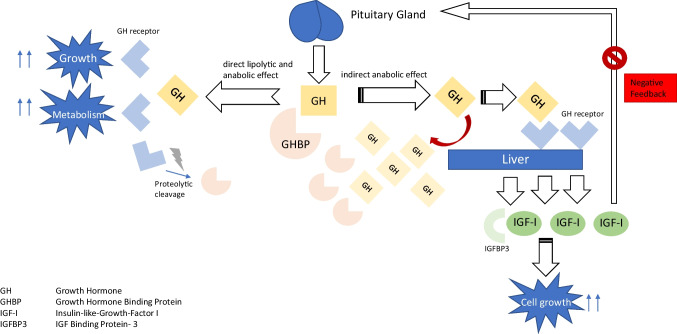
The GH-IGF-I axis (simplified); a simplified version of the human GH-IGF-I axis. GH, growth hormone; GHBP, growth hormone binding protein; IGF-I, insulin-like growth factor I; IGFBP3, IGF binding protein-3

Cardiac wasting–associated cardiomyopathy has recently been described in patients with advanced-stage cancer [1, 2] and was shown to be associated with reduced physical performance, increased all-cause mortality, and inflammation. In preclinical models, the loss of left ventricular (LV) mass has been attributed to cytokine-mediated inflammatory processes and malnutrition [2, 3]. The pathophysiological process in humans causing a remodelling process in the heart as well as an absolute decline of heart muscle tissue remains to be established. Chronic illnesses such as heart failure (HF) and cancer are often characterized by enhanced catabolism and malnutrition predisposing to loss of skeletal muscle mass (sarcopenia) and loss of fat and lean muscle mass with global weight loss (cachexia). The GH-IGF-I axis is an important regulator of the wasting processes in chronic heart failure [4–7]. The GH-IGF-I axis consists of a complex interplay of several stages in this hormonal system (Fig. 1) [5, 8]. Growth hormone (GH) is periodically released from the pituitary gland and leads to the activation of both lipolysis and anabolic effects in the body, affecting men and women similarly. GH’s main anabolic mediator is insulin-like growth factor I (IGF-I) that is mainly bound to IGF-Binding Protein 3 (IGFBP3) when circulating in the blood. GH itself is an important anabolic hormone that mediates several cardiometabolic processes but requires an adequate IGF-I response. Growth hormone binding protein (GHBP) is a surrogate marker for the amount of cellular GH receptors. Derived by proteolytic cleavage of the extracellular binding domain of the GH receptor, GHBP is structurally identical to the GH receptor ectodomain, and therefore both (GH receptor and GHBP) are in constant competition for GH. Depending on feedback mechanisms, GHBP can then either prolong the efficacy of GHBP-bound GH or exclude GH from binding to the GH receptor. In patients with chronic HF and GH treatment, IGF-I has been shown to positively correlate with LV mass [9]. Acquired GH resistance (as seen in cachectic HF patients) [10] is characterized by a shortage of cellular GH receptors (i.e., GHBP is reduced), elevated circulating GH, and low IGF-I [8, 11]. GH sensitivity, as represented by the IGF-I/GH ratio, is very important as isolated IGF-I levels alone cannot be interpreted. We hypothesized that GH resistance is also important in the development of cardiac wasting and designed this study to test this hypothesis in cancer patients.

## Methods

### Patient population

In a prospective single-center study, we enrolled 307 cancer patients (159 female, 148 male, advanced stage III/IV 83%) hospitalized at the oncology wards of Charité – Universitätsmedizin Berlin, Campus Benjamin Franklin and Campus Virchow-Klinikum, with assessment of GH-IGF-I biochemical status. Of 332 prospectively examined cancer patients, we excluded 12 patients due to newly diagnosed cardiac dysfunction and 13 patients due to inadequate imaging quality that precluded assessment of LV mass. Recruitment took place between September 2017 and October 2020. At baseline, all patients where (A) ≥ 18 years of age, (B) had a histologically confirmed active cancer disease, and (C) had no other diagnosis of cancer in the previous 5 years. All patients provided written informed consent for participation in the study.

We excluded cancer patients with (A) significant cardiovascular (CV) disease such as myocardial infarction, coronary artery disease, major valvular defects, or a left ventricular ejection fraction (LVEF) < 50% at baseline; (B) presence of acute, antibiotic-treated infection, and (C) all patients with chronic obstructive pulmonary disease GOLD stages ≥ 3 [12] were excluded from participation in the study—whereas all lung cancer patients could participate in the study, regardless of GOLD stage. The presence of uncomplicated type II diabetes mellitus (T2DM) and controlled arterial hypertension (RR < 160/100 mmHg) were not reasons for exclusion. Cancer entities are displayed in Table S3.

### Study design

We assessed patients with mostly advanced cancer stage. Definition for advanced cancer stage was Union for International Cancer Control (UICC) [13] stage III and IV, the Ann Arbor [14] classification stage III and IV, and Durie and Salmon classification [15] stage III. The possibility of a follow-up examination was provided to every study participant preferably after 3–6 months (maximum 12 months) after baseline assessment. All cancer patients were grouped in tertiles of LV mass adjusted for height squared (height^2^) using sex-specific cut-offs [1, 16]. Additionally, cancer patients were grouped according to the presence/absence of wasting syndrome with unintentional weight loss at baseline defined as body mass index (BMI) < 24.0 kg/m^2^ and weight loss of at least 5% during the previous 12 months [17–19]. The study was approved by the Ethics Committee of the Charité and conducted in accordance with the Declaration of Helsinki.

### Echocardiography and LV mass

Echocardiographic examination and analysis were performed by three experienced echocardiographers in minimum dual control principle with a Vivid E90 echocardiography device (GE, Boston, USA) utilizing Tomtec analyzing software (Unterschliessheim, Germany). To calculate LV mass, we applied the Devereux [20] formula, using the linear measurements of left ventricular wall thickness and left ventricular diameter at end-diastole in parasternal long axis view. All echocardiographic variables are shown as absolute values or adjusted for height [2, 21].

### Blood sampling

We collected venous blood samples of the patients in the mornings. Hormonal parameters of the GH-IGF-I axis were analyzed using ELISA: growth hormone (Roche, assay range 30–50.000 pg/mL inter coefficient of variation (CV)%, 2.23–4.73), growth hormone binding protein (R&D Systems, assay range, 125–80.000 pg/mL, inter CV%, 4.53–6.78), IGFBP3 (Roche, assay range, 0–5000 ng/mL, inter CV%, 8.9–10.66), IGF-I (Roche, assay range, 0–4.0 ng/mL, inter CV%, 3.54–8.31).

### Statistical analyses

To determine normal distribution of variables, we used the Kolmogorov–Smirnov test. To assess between-group differences, Fisher post hoc tests and one-way analysis of variance were used. We presented mean ± standard error (SEM) for normally distributed values when appropriate. For non-normally distributed variables, we displayed the data as median and interquartile range (IQR) and used the Mann–Whitney *U* test as well as the Kruskal–Wallis test, as appropriate. The chi-square test was used to compare frequencies. We calculated the best cut-off to predict LV mass change during follow-up for IGF-I using ROC analysis.

A *p*-value < 0.05 was considered statistically significant in all analyses. Analyses for this paper were generated using SAS/STAT software version 9.4 (SAS Institute, Inc), Stata (StataCorp. 2021, Stata Statistical Software: Release 17, College Station, TX: StataCorp LLC.), and SPSS software version 26.0 (IBM Corp).

## Results

### Baseline characteristics

A total of 307 patients with mostly advanced-stage cancer were included in this study (52% female, mean age 62 ± 1 years (SEM)), mean BMI was 24.6 ± 0.3 kg/m^2^ (SEM), and 152 (50%) were cachectic. Baseline characteristics are displayed in Tables 1 and 2.

**Table 1:** Baseline characteristics of women with advanced-stage cancer

Measurement	All women *n* = 159	LV mass/height^2^ < 50.00 g/m^2^ (1^st^ tertile, *n* = 53)	LV mass/height^2^ ≥ 50.00– < 61.15 g/m^2^ (2^nd^ tertile, *n* = 53)	LV mass/height^2^ ≥ 61.15 g/m^2^ (3^rd^ tertile, *n* = 53)	*p*-value, ANOVA
Clinical variables					
Age (years)	62 ± 1.1	53 ± 2.0###, ***	64 ± 1.6	68 ± 1.6	** < 0.001**
Body mass index, BMI (kg/m^2^)	24.1 ± 0.4	21.8 ± 0.6##, ***	24.5 ± 0.7	26.1 ± 0.7	** < 0.001**
Wasting syndrome with unintentional weight loss, *n* (%)	85 (54)	37 (70)***	29 (55)*	19 (36)	**0.002**
Markers of the GH-IGF-I axis					
Growth hormone, GH (ng/mL)	0.97 (0.35–2.55)	1.82 (0.86–4.17)#, *	0.82 (0.21–2.18)	0.51 (0.27–1.63)	**0.029**
Insulin-like growth factor-I, IGF-I (ng/mL)	64.7 (40.7–97.4)	67.6 (48.2–115.9)**	71.6 (47.9–95.3)*	48.5 (34.9–75.5)	**0.007**
Log IGF-I/GH ratio	1.83 ± 0.05	1.60 ± 0.09##, *	2.02 ± 0.09	1.88 ± 0.09	**0.004**
Growth hormone binding protein, GHBP (pmol/L)	384 (138–663)	276 (144–627)	294 (132–696)	424 (125–685)	0.54
IGF-I binding protein 3, IGFBP3 (µg/mL)	1.82 ± 0.05	1.97 ± 0.09**	1.90 ± 0.08*	1.65 ± 0.08	**0.022**
Cancer and anti-cancer therapy details					
Cancer stage III/IV, *n* (%)	127 (79)	41 (77)	45 (85)	41 (77)	0.56
Solid cancer, *n* (%)	98 (62)	31 (58)	39 (74)	28 (53)	0.08
ECOG Performance Scale points	1.96 ± 0.09	1.68 ± 0.14	2.09 ± 0.16	2.09 ± 0.16	0.09
Karnofsky Index (%)	70.8 ± 1.6	74.5 ± 2.4	66.7 ± 2.9	69.1 ± 2.8	0.24
Systemic anti-cancer therapy naïve, *n* (%)	25 (16)	7 (13)	6 (11)	12 (23)	0.23
Cancer drugs causing heart failure (ESC guidelines ‘22), *n* (%)	66 (42)	22 (42)	23 (43)	21 (40)	0.98
Cancer therapies associated with cardiomyopathy (AHA ‘22), *n* (%)	88 (55)	29 (55)	32 (60)	27 (51)	0.62
Side diagnosis					
Anemia, *n* (%)	109 (69)	36 (68)	31 (58)	42 (79)	0.08
Arterial hypertension, *n* (%)	79 (50)	14 (26)##, ***	29 (55)	36 (68)	** < 0.001**
Hypercholesterolemia, *n* (%)	49 (31)	8 (15)##, *	23 (43)	18 (34)	**0.004**
Type II diabetes mellitus, *n* (%)	20 (13)	1 (2)#, **	8 (15)	11 (21)	**0.008**
Chronic kidney disease, *n* (%)	9 (6)	1 (2)	3 (6)	5 (9)	0.30
Medication					
ACE inhibitors/ARBs, *n* (%)	41 (26)	8 (15)	16 (30)	17 (32)	0.09
Beta-blockers, *n* (%)	28 (18)	6 (11)	9 (17)	13 (25)	0.22
Anticoagulants, *n* (%)	6 (4)	2 (4)	2 (4)	2 (4)	1.00
Diuretics, *n* (%)	30 (19)	3 (6)***	10 (19)	17 (32)	**0.003**
Antidepressants, *n* (%)	25 (16)	11 (21)	8 (15)	6 (11)	0.45
Opioids, *n* (%)	43 (27)	9 (17)	19 (36)	15 (28)	0.08
Corticosteroids, *n* (%)	46 (29)	12 (23)	18 (34)	16 (30)	0.47

vs. 2^nd^ tertile: # < 0.05, ## < 0.01, ### < 0.001

vs. 3^rd^ tertile: * < 0.05, ** < 0.01, *** < 0.001

*ACE*, angiotensin-converting enzyme; *ARB*, angiotensin II receptor blocker; *BSA*, body surface area; *ECOG*, Eastern Co-operative of Oncology Group [22]; Karnofsky Index [23]; *ESC*, European Society of Cardiology cardio-oncology guidelines 2022 [24]; *AHA*, American Heart Association 2022 AHA/ACC/HFSA Guideline for the Management of Heart Failure [25]; ANOVA *p*-value/chi-squared (*χ*^2^) test for comparison between LV mass-tertile 1, LV mass-tertile 2, and LV mass-tertile 3 of female cancer patients. Normal distributed variables are presented as means ± SEM, non-normally distributed variables as median (interquartile range) and nominal variables as *n* (%), *p*-values <0.05 are bold

**Table 2:** Baseline characteristics of men with advanced-stage cancer

Measurement	All men *n* = 148	LV mass/height^2^ < 59.58 g/m^2^ (1^st^ tertile, *n* = 48)	LV mass/height^2^ ≥ 59.58– < 74.06 g/m^2^ (2^nd^ tertile, *n* = 51)	LV mass/height^2^ ≥ 74.06 g/m^2^ (3^rd^ tertile, *n* = 49)	*p*-value, ANOVA
Clinical variables					
Age (years)	62 ± 1.1	63 ± 2.4	61 ± 1.8	63 ± 1.6	0.53
Body mass index, BMI (kg/m^2^)	25.1 ± 0.4	22.4 ± 0.5###, ***	26.4 ± 0.6	26.4 ± 0.7	** < 0.001**
Wasting syndrome with unintentional weight loss, *n* (%)	67 (45)	32 (67)##, **	17 (33)	18 (37)	**0.001**
Markers of the GH-IGF-I axis					
Growth hormone, GH (ng/mL)	0.91 (0.26–2.34)	2.11 (0.77–3.22)##, **	0.56 (0.12–1.65)	0.70 (0.19–1.89)	**0.003**
Insulin-like growth factor-I, IGF-I (ng/mL)	73.6 (46.6–107.2)	73.4 (49.6–108.8)	78.3 (51.8–113.4)	68.9 (42.6–107.7)	0.98
Log IGF-I/GH ratio	1.95 ± 0.06	1.64 ± 0.09###, **	2.14 ± 0.11	2.04 ± 0.11	**0.002**
Growth hormone binding protein, GHBP (pmol/L)	325 (108–857)	413 (159–972)	233 (95–598)	293 (89–950)	0.44
IGF-I binding protein 3, IGFBP3 (µg/mL)	1.45 ± 0.05	1.41 ± 0.08	1.49 ± 0.08	1.47 ± 0.08	0.75
Cancer and anti-cancer therapy details					
Cancer stage III/IV, *n* (%)	127 (86)	43 (90)	44 (86)	40 (82)	0.54
Solid cancer, *n* (%)	72 (49)	28 (58)	18 (35)	26 (53)	0.05
ECOG Performance Scale points	1.49 ± 0.09	1.54 ± 0.17	1.25 ± 0.15	1.67 ± 0.17	0.18
Karnofsky Index (%)	76.2 ± 1.6	72.7 ± 3.0	81.6 ± 2.5	73.9 ± 2.8	0.05
Systemic anti-cancer therapy naïve, *n* (%)	16 (11)	7 (15)	5 (10)	4 (8)	0.55
Cancer drugs causing heart failure (ESC guidelines ‘22), *n* (%)	72 (49)	27 (56)	23 (45)	22 (45)	0.46
Cancer therapies associated with cardiomyopathy (AHA ‘22), *n* (%)	99 (67)	36 (75)	35 (69)	28 (57)	0.17
Side diagnosis					
Anemia, *n* (%)	113 (76)	42 (86)##	32 (63)	39 (80)	**0.014**
Arterial hypertension, *n* (%)	65 (44)	21 (44)	19 (37)	25 (51)	0.39
Hypercholesterolemia, *n* (%)	23 (16)	5 (10)	7 (14)	11 (22)	0.26
Type II diabetes mellitus, *n* (%)	23 (16)	7 (15)	8 (16)	8 (16)	1.00
Chronic kidney disease, *n* (%)	12 (8)	2 (4)	3 (6)	7 (14)	0.17
Medication					
ACE inhibitors/ARBs, *n* (%)	37 (25)	9 (19)	11 (22)	17 (35)	0.17
Beta-blockers, *n* (%)	25 (17)	9 (19)	6 (11)	10 (21)	0.48
Anticoagulants, *n* (%)	6 (4)	1 (2)	0	5 (10)	**0.023**
Diuretics, *n* (%)	26 (18)	8 (17)	7 (14)	11 (22)	0.55
Antidepressants, *n* (%)	17 (12)	6 (13)	6 (12)	5 (10)	0.95
Opioids, *n* (%)	28 (19)	11 (23)	5 (10)	12 (25)	0.11
Corticosteroids, *n* (%)	55 (37)	22 (46)	19 (37)	14 (29)	0.23

vs. 2^nd^ tertile: # < 0.05, ## < 0.01, ### < 0.001

vs. 3^rd^ tertile: * < 0.05, ** < 0.01, *** < 0.001

*ACE*, angiotensin-converting enzyme, *ARB*, angiotensin II receptor blocker; *BSA*, body surface area; *ECOG*, Eastern Co-operative of Oncology Group; *ESC*, European Society of Cardiology cardio-oncology guidelines 2022; *AHA*, American Heart Association 2022 AHA/ACC/HFSA Guideline for the Management of Heart Failure; ANOVA *p*-value/chi-squared (*χ*^2^) test for comparison between LV mass-tertile 1, LV mass-tertile 2, and LV mass-tertile 3 of male cancer patients. Normal distributed variables are presented as means ± SEM, non-normally distributed variables as median (interquartile range), and nominal variables as *n* (%), *p*-values <0.05 are bold

### Group stratification by left ventricular mass

**Fig. 2:**
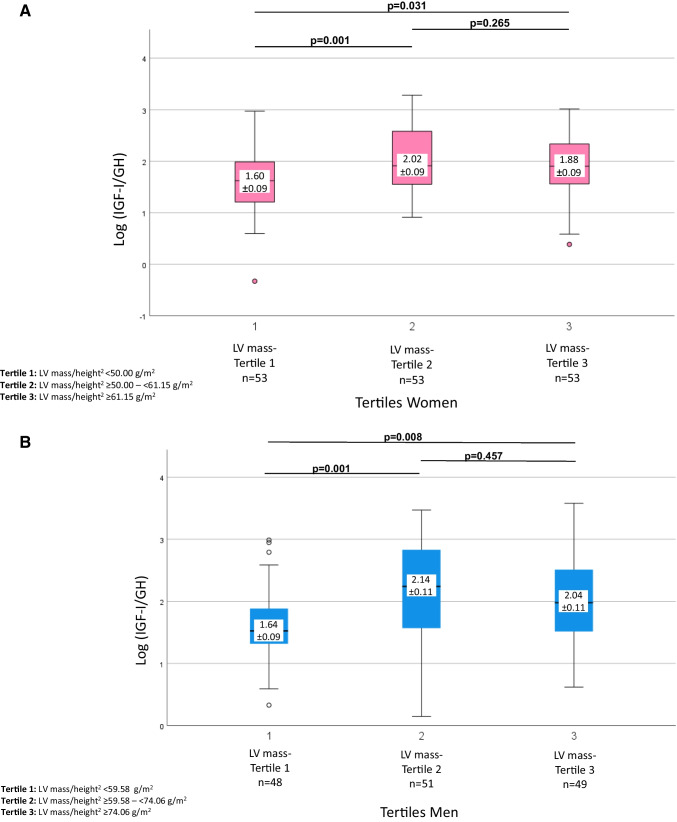
**A** LV mass according to tertiles of log(IGF-I/GH) in women; **A** compares the ratio of Log (IGF-I/GH) in dependence of LV mass adjusted for height squared tertiles in women. GH, growth hormone; IGF-I, insulin-like growth factor I; Log (IGF-I/GH), log insulin-like growth factor I/growth hormone ratio; LV, left ventricular. **B** LV mass according to tertiles of log(IGF-I/GH) in men; **B** compares the ratio of Log (IGF-I/GH) in dependence of LV mass adjusted for height squared tertiles in men. GH, growth hormone; IGF-I, insulin-like growth factor I; Log (IGF-I/GH), log insulin-like growth factor I/growth hormone ratio; LV, left ventricular

**Fig. 3:**
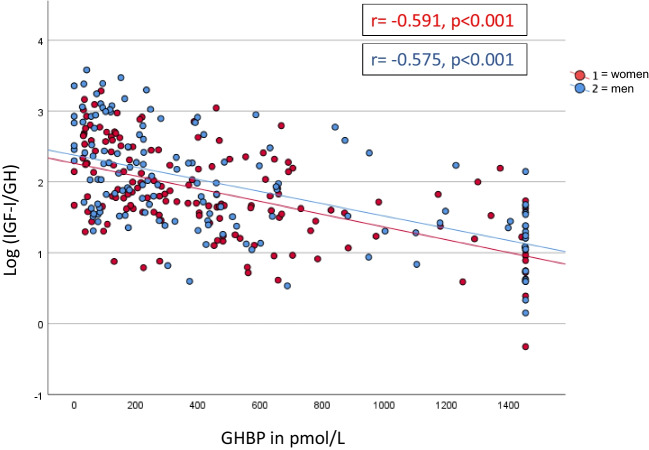
Relationship between ratio of insulin-like growth factor-I (IGF-I) to growth hormone (GH) (log IGF-I/GH ratio) vs. growth hormone binding protein (GHBP in pmol/L) in patients with cancer; displays the correlation of Log (IGF-I/GH) ratio with the growth hormone binding protein in our cohort. GH, growth hormone; GHBP, growth hormone binding protein; IGF-I, insulin-like growth factor I; Log (IGF-I/GH), log insulin-like growth factor I/growth hormone ratio

Patients were grouped by LVmass/height^2^ tertiles, as assessed by transthoracic echocardiography (tertiles females, < 50.00 g/m^2^, 50.00– < 61.15 g/m^2^, ≥ 61.15 g/m^2^; tertiles males, < 59.85 g/m^2^, 59.85– < 74.06 g/m^2^, ≥ 74.06 g/m^2^; Tables 1 and 2). Patients in the lowest tertile were younger, had a lower BMI, and more frequently demonstrated wasting syndrome with unintentional weight loss. Cancer stage, cardiotoxic anti-cancer therapies [24, 25], and presence of solid cancer were similar in all three groups. Cancer patients in the lowest tertile showed higher levels of GH and lower log IGF-I/GH ratio (Fig. 2A, B). LV mass is significantly associated with the IGF-I/GH ratio (Pearson correlation coefficient, 0.203, *p* < 0.001). In a multivariate model, markers of the GH-IGF-I axis are significant predictors for the LV mass (*R*^2^ = 0.079; Beta(IGF-I) =  − 0.101, *p* = 0.12; Beta(GH) =  − 0.193, *p* < 0.001; Beta(IGF-I/GH) = 0.167, *p* = 0.007; Beta(GHBP) = 0.143, *p* = 0.04). Among females, lower LV mass was associated with higher levels of IGF-I, whereas such association did not exist for males. GHBP and IGFBP3 were not associated with reduced LV mass, whereas in all cancer patients, GHBP negatively correlated with log IGF-I/GH ratio (Fig. 3). When adjusting for age (log(IGF-I/GH)/age ratio vs. GHBP), the correlation remained significant (female, *p* < 0.001; male, *p* < 0.001).

### Group stratification by wasting syndrome with unintentional weight loss

Women and men were stratified by presence or absence of wasting syndrome with unintentional weight loss and baseline characteristics are provided in Table 3. Cachectic cancer patients in general less frequently had hypercholesterinemia, T2DM, and arterial hypertension. Patients with vs. without wasting syndrome with unintentional weight loss had higher GH and GHBP, lower log IGF-I/GH ratio, and similar levels of IGF-I and IGFBP3. Cancer patients with wasting syndrome with unintentional weight loss had lower LV mass and LV mass/height^2^ than patients without. There was no difference in cancer stage or administration of cardiotoxic anti-cancer therapies regarding presence of wasting syndrome with unintentional weight loss.

**Table 3:** Baseline characteristics of cachectic and non-cachectic women (*n* = 159) and men (*n* = 148) (wasting syndrome with unintentional weight loss defined as weight loss ≥ 5% in the last 12 months and BMI < 24 kg/m^2^)

Measurement	All patients *n* = 307	Women with wasting syndrome with unintentional weight loss *n* = 85	Women without wasting syndrome with unintentional weight loss *n* = 74	*p*-value	Men with wasting syndrome with unintentional weight loss *n* = 67	Men without wasting syndrome with unintentional weight loss *n* = 81	*p*-value
Clinical variables							
Age (years)	62 ± 0.8	61 ± 1.6	63 ± 1.6	0.43	62 ± 1.9	63 ± 1.4	0.92
Body mass index, BMI (kg/m^2^)	24.6 ± 0.3	20.5 ± 0.3	28.3 ± 0.5	** < 0.001**	21.3 ± 0.3	28.3 ± 0.4	** < 0.001**
Cancer and anti-cancer therapy details							
Cancer stage III/IV, *n* (%)	254 (83)	71 (84)	56 (76)	0.24	58 (87)	69 (85)	0.82
Solid cancer, *n* (%)	170 (55)	54 (64)	44 (60)	0.63	41 (61)	31 (38)	**0.008**
ECOG Performance Scale points	1.73 ± 0.07	2.18 ± 0.13	1.70 ± 0.12	**0.008**	1.81 ± 0.16	1.22 ± 0.11	**0.002**
Karnofsky Index (%)	73.4 ± 1.1	66.6 ± 2.3	75.5 ± 1.9	**0.005**	70.3 ± 2.8	80.9 ± 1.7	**0.001**
Systemic anti-cancer therapy naïve, *n* (%)	41 (13)	12 (14)	13 (18)	1.00	5 (8)	11 (14)	0.59
Cancer drugs causing heart failure (ESC guidelines ‘22), *n* (%)	138 (45)	38 (45)	28 (38)	0.42	31 (46)	41 (51)	0.60
Cancer therapies associated with cardiomyopathy (AHA ‘22), *n* (%)	187 (61)	52 (61)	36 (49)	0.11	45 (67)	54 (67)	0.95
Laboratory variables							
Growth hormone, GH (ng/mL)	0.94 (0.27–2.42)	1.45 (0.49–3.78)	0.54 (0.25–1.63)	**0.029**	1.79 (0.66–3.28)	0.53 (0.17–1.88)	** < 0.001**
Insulin-like growth factor-I, IGF-I (ng/mL)	69.2 (44.3–100.3)	63.6 (43.9–95.3)	68.4 (37.4–98.2)	0.56	69.0 (47.2–112.3)	80.7 (50.6–107.1)	0.37
Log IGF-I/GH ratio	1.89 ± 0.04	1.68 ± 0.08	2.00 ± 0.07	**0.003**	1.68 ± 0.09	2.15 ± 0.07	** < 0.001**
Growth hormone binding protein, GHBP (pmol/L)	331 (128–668)	444 (194–760)	272 (64–640)	**0.038**	431 (220–1455)	216 (84–470)	** < 0.001**
IGF-I binding protein 3, IGFBP3 (µg/mL)	1.66 ± 0.04	1.85 ± 0.07	1.83 ± 0.08	0.86	1.45 ± 0.07	1.47 ± 0.06	0.87
Side diagnosis							
Anemia, *n* (%)	222 (72)	65 (77)	44 (60)	**0.026**	56 (84)	57 (70)	0.08
Arterial hypertension, *n* (%)	144 (47)	31 (37)	48 (65)	**0.001**	24 (36)	41 (51)	0.09
Hypercholesterolemia, *n* (%)	72 (24)	23 (27)	26 (35)	0.30	6 (9)	17 (21)	0.07
Type II diabetes mellitus, *n* (%)	43 (14)	6 (7)	14 (19)	**0.032**	7 (10)	16 (20)	0.17
Chronic kidney disease, *n* (%)	21 (7)	2 (2)	7 (10)	0.08	5 (8)	7 (9)	1.00
Medication							
ACE inhibitors/ARBs, *n* (%)	78 (25)	16 (19)	25 (34)	**0.045**	10 (15)	27 (33)	**0.013**
Beta-blockers, *n* (%)	53 (17)	8 (9)	20 (27)	**0.006**	9 (13)	16 (20)	0.38
Anticoagulants, *n* (%)	12 (4)	0	6 (8)	**0.009**	3 (5)	3 (4)	1.00
Diuretics, *n* (%)	56 (18)	9 (11)	21 (28)	**0.005**	8 (12)	18 (22)	0.13
Antidepressants, *n* (%)	42 (14)	14 (17)	11 (15)	0.83	6 (9)	11 (14)	0.45
Opioids, *n* (%)	71 (23)	23 (27)	20 (27)	1.00	16 (24)	12 (15)	0.21
Corticosteroids, *n* (%)	101 (33)	20 (24)	26 (35)	0.12	23 (34)	32 (40)	0.61
Echocardiographic variables							
Left ventricular (LV) mass (g)	183 ± 3	144 ± 5	169 ± 4	** < 0.001**	195 ± 7	225 ± 5	** < 0.001**
LV mass adjusted to height^2^ (g/m^2^)	62 ± 1	53 ± 2	62 ± 2	** < 0.001**	62 ± 2	71 ± 2	** < 0.001**

*ACE*, angiotensin-converting enzyme, *ARB*, angiotensin II receptor blocker; *BSA*, body surface area; *ECOG*, Eastern Co-operative of Oncology Group; *ESC*, European Society of Cardiology cardio-oncology guidelines 2022; *AHA*, American Heart Association 2022 AHA/ACC/HFSA Guideline for the Management of Heart Failure; *t*-test *p*-value/chi-squared (*χ*^2^) test for comparison between patients with wasting syndrome with unintentional weight loss vs. without. Normal distributed variables are presented as means ± SEM, non-normally distributed variables as median (interquartile range), and nominal variables as *n* (%), *p*-values <0.05 are bold

### Follow-up analysis

**Fig. 4:**
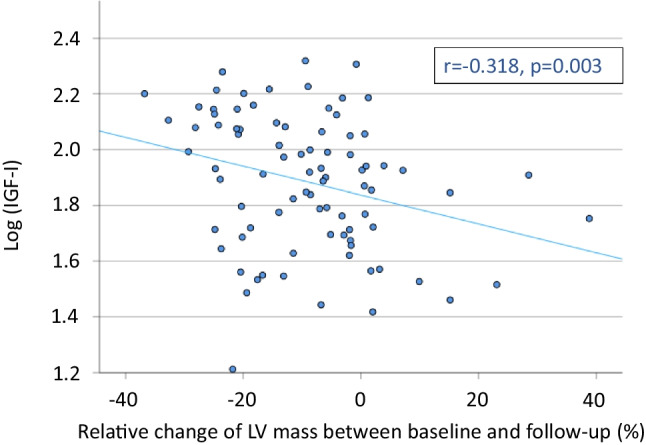
Correlation of relative change in LV mass (%) vs. insulin-like growth factor-I (log(IGF-I)) at baseline in 85 patients with cancer with follow-up; the change of LV mass over time of 85 patients with cancer in dependence of IGF-I levels at baseline. Log (IGF-I/GH), log insulin-like growth factor I/growth hormone ratio; LV, left ventricular

Eight-five cancer patients (41 female, 44 male) had a follow-up assessment after a mean of 153 ± 16 days. During that time, LV mass declined by on average − 18.8 ± 2.6 g (i.e., − 9.2 ± 1.5% [SEM]). Thirty-nine patients (46%) had a reduction of LV mass by ≥ 10%. Higher IGF-I baseline values were associated with a reduction of LV mass at follow-up (univariate *r* =  − 0.318, *p* = 0.003; multiple linear regression model standardized coefficient (Beta) − 0.308, *p* = 0.02—adjusted for sex, age, BMI, and GHBP, Table S1/Fig. 4). Best IGF-I cut-off for LV mass change prediction was ≥ 98.22 ng/mL. Patients with lower vs. higher IGF-I levels were older, had a similar BMI and frequency of wasting syndrome with unintentional weight loss, higher LV mass at baseline and GHBP levels, lower IGFBP3 and IGF-I/GH ratio, and similar levels of GH (Table S2). Further, when adjusting for age, the log (IGF-I)/age ratio vs. relative change of LV mass (%) significantly correlate (*r* =  − 0.314, *p* = 0.003).

## Discussion

This analysis provides new insights into the regulation of the GH-IGF-I axis with regard to the LV mass of cancer patients. We found that reduced LV mass (i.e., cardiac wasting) in patients with mostly advanced-stage cancer is associated with an imbalance in the GH-IGF-I axis. Low LV mass was associated with higher GH levels (with mostly stable GHBP and IGF-I levels observed across LV mass tertiles) and lower log IGF-I/GH ratio, indicating a state of acquired GH resistance in these patients. Overcoming GH resistance therapeutically may be beneficial for cancer patients with cardiac wasting–associated cardiomyopathy.

Cardiac wasting is known to occur in patients with chronic HF [26] and is attributed to chronic inflammatory processes, oxidative and metabolic stress, as well as severe malnutrition. Patients with advanced-stage cancer show similar systemic metabolic abnormalities as chronic HF patients marked by severe cachexia [27–29] and suffer from cardiac wasting without overt LV dysfunction, at least when standard criteria like LVEF are considered [1]. Previous studies have demonstrated a link between imbalance in the GH-IGF-I axis and worsening cachexia in patients with advanced-stage cancer [30]. The current study found an inverse relationship between LV mass and circulating GH levels and IGF-I/GH ratio indicative of an acquired GH resistance. GHBP levels were consistent across all LV mass tertiles in men and women. Interestingly, increased levels of markers of the GH-IGF-I axis have previously been linked to incidence of breast cancer [31]. IGF-I is found to promote tumor growth [32–34] and prevent cell apoptosis [35, 36], including skeletal muscle cells. GH levels are also known to be much higher in untreated vs. treated heart failure patients [37]. These findings suggest that GH resistance is present in cancer patients with cardiac wasting and may potentially contribute towards cardiac wasting–associated cardiomyopathy, although a causal relationship remains to be determined.

We stratified our findings based on sex to account for differences in baseline LV mass and potential effects of sex-specific endocrine systems. Women were found to have significantly lower levels of GH and IGF-I with increased LV mass. A variable GH-IGF-I axis response has been reported previously in the setting of hormone replacement therapy in women and men [38]. No significant differences in GHBP levels were found across LV mass tertiles among men or women. Among men, a similar trend was observed between GH and LV mass tertiles; however, no significant difference in IGF-I levels was observed for men across the LV mass spectrum. These findings of different IGF-I patterns in men and women may be attributed to inherent sex-specific differences in responsiveness to GH levels [38]. Additionally, high IGF-I levels at baseline were a predictor for LV mass loss at follow-up. Several studies have reported reduced sensitivity of exogenous GH analogue supplementation in stimulating circulating IGF-I in GH-deficient women compared to men [39]. It can be reasonably inferred that the variable trends in IGF-I levels with increasing GH levels in women across LV mass tertiles may not be of special cancer-specific clinical significance as levels predictably remained lower than those observed in men. Previous studies in gastrointestinal cancer, for instance, reported low GH levels and low log IGF-I/GH ratio in combination with normal IGF-I levels [5, 30, 40]. Consequently, the reported normal IGF-I levels but high GH levels for our study suggest presence of a secondary catabolic state and point towards an independent loss of LV mass in the context of whole body wasting. This clinical scenario may also resemble untreated heart failure. [2]

We found a high prevalence of wasting syndrome with unintentional weight loss across all LV mass tertiles, with increasing frequency of wasting syndrome with unintentional weight loss with worsening LV mass across both sexes. Since the link between cachexia and acquired GH resistance has previously been established [7, 10, 41], we performed a secondary analysis where patients were classified by the presence or absence of wasting syndrome with unintentional weight loss to assess the GH-IGF-I axis in patients with vs. without. We found that men and women with wasting syndrome with unintentional weight loss had significantly higher levels of GH and GHBP and lower IGF-I/GH ratio compared to men and women without wasting syndrome with unintentional weight loss, with no significant differences between IGF-I levels. Wasting syndrome with unintentional weight loss leads to a reactionary increase in GH and if IGF-I fails to respond the wasting gets worse—therefore, it is hard to interpret an IGF-I level without knowing the GH level first.

Moreover, patients with wasting syndrome with unintentional weight loss also had significantly lower LV mass compared with patients without. This can potentially be explained by a greater degree of progression of GH resistance in patients who have developed wasting syndrome with unintentional weight loss, compared to patients without. Moreover, acquired GH resistance has been associated with age-mediated loss of skeletal muscle mass (i.e., primary sarcopenia) in geriatric patients [42]. These findings suggest that cancer-related wasting syndrome with unintentional weight loss and cardiac wasting, although modulated by similar mechanisms, can occur independently of each other, or occur sequentially after each other [43, 44].

We excluded patients with active, antibiotic-treated infection to reduce the possible catabolic effects of an acute systemic immune response influencing the GH-IGF-I axis [45]. However, chronic inflammation is persistent and progressive in patients with advanced-stage cancer and is known to contribute towards wasting processes [46]. It is still to be determined if inflammation predates the development of GH resistance. However, here we show that blunting of GH’s anabolic activity with GH resistance is associated with loss of LV mass (i.e., cardiac wasting).

Anabolic interventions—specifically in the context of cardiac wasting–associated cardiomyopathy of cancer patients or as part of cancer cachexia therapies [47, 48]—could provide such direct evidence. Clinical trials in advanced cancer and cancer cachexia per se should monitor cardiac effects of these interventions in detail.

In recent studies conducted by our group, we investigated the presence of cardiac cachexia in advanced-stage cancer patients and the risk of developing a HF-like syndrome in the setting of cardiac wasting–associated cardiomyopathy [1, 2, 49]; however, studies have been limited in the assessment of the underlying mechanisms and prognostic value of already known risk factors. As described earlier, higher levels of circulating IGF-I levels have been associated with improved myocardial mass with GH supplementation in patients with chronic HF, although trial results have been variable in terms of improvement in hard clinical endpoints [9, 50, 51]. Cardiac wasting–associated cardiomyopathy is a separate entity that may behave differently to augmentation of the GH-IGF-I axis, but that remains to be proven. Alterations in GH-IGF-I axis observed with changes in LV mass may serve as a sensitive screening tool for earlier detection of cardiac wasting–associated cardiomyopathy in patients with cancer to allow for earlier implementation of appropriate treatment measures.

### Study limitations

This was a prospective cross-sectional study that enrolled patients from one center using standardized assessments. Still, causality cannot be inferred from those studies. Although we found no significant difference in LV mass loss in anti-cancer treatment naïve and treated patients, it might be desirable to study whether specific anti-cancer therapies could influence LV mass. We did not exclude patients with controlled arterial hypertension nor T2DM in an intention to show a real-world cohort of cancer patients—but those patients could be excluded in future studies. We excluded all patients with significant cardiovascular disease at baseline with the intent to not bias the analyses by such underlying diseases—further studies in the future could therefore exclusively investigate those patients with cancer that already demonstrate significant cardiovascular disease. While our study monitored the longitudinal change of LV mass over time by echocardiography, we did not measure longitudinal changes in GH-IGF-I axis over time, and therefore this could be of great interest for future studies.

## Conclusion

Low LV mass in patients with advanced-stage cancer is associated with an impaired state of the GH-IGF-I axis in both men and women, indicating presence of acquired GH resistance in many of these patients with cardiac wasting. These observations provide further insights into the underlying mechanism of a cancer-related wasting syndrome with unintentional weight loss, and particularly cardiac wasting in patients with cancer, which can result in a cardiomyopathy, leading to impaired exercise capacity and worsening symptoms and quality of life. Further prospective studies are warranted to ascertain a potential causal relationship between acquired GH resistance and cardiac wasting, and to explore related interventions to center-act or prevent cardiac wasting–associated cardiomyopathy in advanced cancer.

## Supplementary Information

Below is the link to the electronic supplementary material.

Supplementary file1 (DOCX 22 KB)
