# Supplementary material for: Association of an impaired GH-IGF-I axis with cardiac wasting in patients with advanced cancer
Source: Clin Res Cardiol. 2024 Apr 8;114(9):1118–32. doi: 10.1007/s00392-024-02400-x (PMC12408708; doi:10.1007/s00392-024-02400-x)
Supplement: Supplementary file 1 — Supplementary file1 (DOCX 22 KB) [file 392_2024_2400_MOESM1_ESM.docx]

**Table S1: Correlation of relative LV mass change (baseline to FU) in % and markers of the GH-IGF-I axis at baseline**

Correlation Relative Change LV Mass vs

| Measurement | r_s_  (95% CI) | *p*-value |
| --- | --- | --- |
| Age (years) | 0.225 (0.036 – 0.411) | **0.040** |
| BMI (kg/m²) | -0.094 (-0.304 – 0.124) | 0.39 |
| GH (ng/mL) | -0.074 (-0.287 – 0.147) | 0.50 |
| IGF-1 (ng/mL) | -0.318 (-0.502 – -0.106)) | **0.003** |
| Log (IGF-1/GH) | -0.048 (-0.265 – 0.173) | 0.66 |
| GHBP (pmol/L) | 0.108 (-0.114 – 0.319) | 0.33 |
| IGFBP3 (µg/L) | -0.178 (-0.381 – 0.042) | 0.10 |

**Table S2: Baseline characteristics of 85 patients with advanced cancer and a follow-up measurement of LV mass, stratified by baseline plasma IGF-I**

| Measurement | IGF-I < 98.22 ng/mL /  Log(IGF-I) < 1.992  n=54 | IGF-I ≥ 98.22 ng/mL /  Log(IGF-I) ≥ 1.992  n=31 | p-value |
| --- | --- | --- | --- |
| Clinical Variables | | | |
| Sex, female, n (%) | 26 (48) | 15 (48) | 1.0 |
| Age (years) | 63 ± 1.9 | 47 ± 2.8 | **<0.001** |
| Body Mass Index, BMI (kg/m²) | 26.0 ± 0.8 | 25.1 ± 0.9 | 0.42 |
| Wasting syndrome with unintentional weight loss, n (%) | 19 (35) | 13 (42) | 0.64 |
| Cancer and anti-cancer therapy details | | | |
| Cancer stage III/IV, n (%) | 38 (70) | 20 (65) | 0.63 |
| Solid cancer, n (%) | 19 (35) | 8 (26) | 0.47 |
| ECOG performance scale points | 1.44 ± 0.16 | 1.06 ± 0.15 | 0.11 |
| Karnofsky Index (%) | 80.4 ± 2.4 | 83.6 ± 2.3 | 0.38 |
| Systemic anti-cancer therapy naïve, n (%) | 8 (15) | 4 (13) | 1.0 |
| Laboratory Variables | | | |
| Growth Hormone, GH (ng/mL) | 0.51 (0.18-1.61) | 0.72 (0.16-2.57) | 0.33 |
| Insulin-like growth factor-1, IGF-1 (ng/mL) | 58.3 (40.6-79.7) | 134.1 (118.0-158.6) | **-** |
| log IGF-1/GH ratio | 2.02 ± 0.09 | 2.33 ± 0.12 | **0.040** |
| Growth hormone binding protein, GHBP (pmol/L) | 319.7 (119.6-516.2) | 82.7 (36.7-224.6) | **0.006** |
| IGF-1 binding protein 3, IGFBP3 (µg/mL) | 1.51 ± 0.07 | 2.11 ± 0.09 | **<0.001** |
| Side diagnosis | | | |
| Anaemia, n (%) | 42 (78) | 22 (71) | 0.60 |
| Arterial Hypertension, n (%) | 23 (43) | 7 (23) | 0.98 |
| Hypercholesterolemia, n (%) | 15 (28) | 9 (29) | 1.0 |
| Type II Diabetes Mellitus, n (%) | 6 (11) | 2 (6) | 0.71 |
| Chronic Kidney Disease, n (%) | 6 (11) | 1 (3) | 0.41 |
| Medication | | | |
| ACE Inhibitors/ARBs, n (%) | 13 (24) | 3 (10) | 0.15 |
| Beta-Blockers, n (%) | 14 (26) | 4 (13) | 0.18 |
| Anticoagulants, n (%) | 1 (2) | 0 (0) | 1.0 |
| Diuretics, n (%) | 10 (19) | 3 (10) | 0.36 |
| Antidepressants, n (%) | 6 (11) | 3 (10) | 1.0 |
| Opioids, n (%) | 7 (13) | 5 (16) | 0.75 |
| Corticosteroids, n (%) | 24 (44) | 10 (32) | 0.36 |
| Echocardiographic Variables | | | |
| Left ventricular (LV) mass (g) | 194 ± 7 | 175 ± 8 | 0.12 |
| LV mass adjusted to height² (g/m²) | 66 ± 2 | 57 ± 2 | **0.015** |

ACE, angiotensin converting enzyme, ARB, angiotensin II receptor blocker; BSA, body surface area; ECOG, Eastern Co-operative of Oncology Group; t-test p-value/ chi-squared (χ²) test for comparison between low log (IGF-1) cancer patients, and higher log (IGF-1) cancer patients. Normal distributed variables are presented as means ± SEM, non-normally distributed variables as median (interquartile range) and nominal variables as n(%).

**Table S3: Cancer types of patients enrolled**

| Cancer diagnosis | Solid / haematological cancer | Number of all patients, n (%) |
| --- | --- | --- |
| Breast cancer | Solid | 39 (13) |
| Colorectal cancer | Solid | 27 (9) |
| Lung cancer | Solid | 27 (9) |
| Pancreatic cancer | Solid | 15 (5) |
| Oral cavity and salivary glands cancer | Solid | 11 (4) |
| Malignant melanoma of choroid | Solid | 13 (4) |
| Prostate cancer | Solid | 10 (3) |
| Oesophagus | Solid | 1 (1) |
| Hepatic-biliary tract cancer | Solid | 2 (1) |
| Stomach | Solid | 1 (1) |
| Gynaecologic cancer (vulva, cervix, fallopian tube, ovary, uterus) | Solid | 6 (2) |
| Soft tissue sarcoma | Solid | 1 (1) |
| Larynx | Solid | 2 (1) |
| Other cancer (major salivary glands, brain, thyroid gland, kidney, malignant melanoma of the skin, unknown) | Solid | 15 (5) |
| Non-Hodgkin lymphoma | Haematological | 92 (30) |
| Classic Hodgkin lymphoma | Haematological | 29 (9) |
| Multiple myeloma | Haematological | 16 (5) |
